# Transcranial Direct Current Stimulation of the Left Dorsolateral Prefrontal Cortex Shifts Preference of Moral Judgments

**DOI:** 10.1371/journal.pone.0127061

**Published:** 2015-05-18

**Authors:** Maria Kuehne, Kai Heimrath, Hans-Jochen Heinze, Tino Zaehle

**Affiliations:** Department of Neurology, Otto-von-Guericke University Magdeburg, Magdeburg, Germany; Ecole Normale Supérieure, FRANCE

## Abstract

Attitude to morality, reflecting cultural norms and values, is considered unique to human social behavior. Resulting moral behavior in a social environment is controlled by a widespread neural network including the dorsolateral prefrontal cortex (DLPFC), which plays an important role in decision making. In the present study we investigate the influence of neurophysiological modulation of DLPFC reactivity by means of transcranial direct current stimulation (tDCS) on moral reasoning. For that purpose we administered anodal, cathodal, and sham stimulation of the left DLPFC while subjects judged the appropriateness of hard moral personal dilemmas. In contrast to sham and cathodal stimulation, anodal stimulation induced a shift in judgment of personal moral dilemmas towards more non-utilitarian actions. Our results demonstrate that alterations of left DLPFC activity can change moral judgments and, in consequence, provide a causal link between left DLPFC activity and moral reasoning. Most important, the observed shift towards non-utilitarian actions suggests that moral decision making is not a permanent individual trait but can be manipulated; consequently individuals with boundless, uncontrollable, and maladaptive moral behavior, such as found in psychopathy, might benefit from neuromodulation-based approaches.

## Introduction

Social cognition describes cognitive mechanisms underlying social behavior, a trait that enables humans to live in social communities [1]. In humans, social behavior is distinguished by an attitude to morality; cultural norms and values define morally right or wrong behavior. Accordingly, social cognition plays a key role when judging about the moral righteousness of motives and actions [2]. Incapacity for moral judgment can lead to severe difficulties in the socialization process, best illustrated in psychopaths. Psychopathy is characterized by diminished guilt and empathy feelings and impulsivity, resulting in deficient moral decision making and inappropriate social behavior [3].

Neurophysiologically, lesion and lobectomy studies revealed that the frontal cortex, especially the prefrontal part, plays an important role in social cognition. Consequently, a dysfunction of this brain region results in disadvantageous social actions and poor judgments (e.g. negative variations in social and emotional decision making, inappropriate behavior, negative changes of mood and of personality) [4, 5]. Current studies on patients with psychopathy [3, 6, 7], antisocial behavior [8], frontotemporal dementia [9], and epilepsy [10] confirm this result and further specify the involved prefrontal brain regions involved in moral and social behavior.

Judgment of moral dilemmas has become a promising method to study the *moral brain*. Here, a moral dilemma is presented as a story involving a person’s moral conflict in taking one of several possible actions [11, 12] justified by competing moral reasons. These reasons are on the one hand given by personal interests and personal moral values and on the other hand by different duties implemented for instance by society or lifestyle. The conflict is based on the impossibility to realize both actions despite convincing reasons [13]. The degree of personal involvement distinguishes between personal and impersonal moral dilemmas. The action in the personal dilemma directly and personally causes harm to another party. The danger of welfare is created and not merely a product of chance [11]. The *Fat Man* problem by Thomson (1985) represents a classical personal moral dilemma. In this case the reader has to imagine himself as walker on a footbridge seeing a runaway trolley that, if not stopped, will kill 5 workmen on the track. A seriously overweight man is standing next to the walker. The body of his weight could stop the train. To push this stranger in front of the train is the only way to save the workmen. In contrast, a classical impersonal dilemma is the trolley problem, introduced by P. Foot (1967). Here the reader has to imagine being the guard of a runaway trolley that is driving directly towards 5 workmen on the track. The only possibility to save these 5 men is to turn the trolley on another track thereby killing only one man who is working on the alternative track [14]. The direct and indirect violation of one’s fundamental right to live draws the difference between these dilemmas. Changing the direction of the train itself is not directly an infringement of the one workman’s right to live. Pushing the man off the bridge, however, directly violates his right to live [15]. Gains and losses in these two dilemmas are the same. The essential difference is given by two different harm variables. The personal moral dilemma causes direct harm whereas for the impersonal moral dilemma harm can be considered as side-effect [13]. According to the dual-process theory, moral decisions are based on both, an automatic (implicit) emotional response and a controlled (explicit) application of utilitarian decision-rules. Being responsible for the death or harm of another person elicits an aversive emotional response, but at the same time, cognitive reasoning favors the utilitarian option. In personal moral dilemmas, the emotional response is assumed to be too strong to be overruled by cognitive processes, whereas in impersonal moral dilemmas the weaker emotional aversion may be subdued by cognitive control—leading to more utilitarian decisions [16].

The emotional and cognitive reasoning processes identified in the dual process theory have been directly related to neurophysiological activity changes and point to the involvement of the DLPFC in moral decision-making [17, 18]. Specifically, activity of the prefrontal cortex is thought to be important for the cognitive reasoning process, which can counteract the emotional response [17]. These fMRI studies demonstrated that when participants had to judge dilemma situations, personal moral dilemmas in comparison to non-moral and impersonal moral dilemmas were associated with greater activations of the frontal cortex—in particular BA 9 and 10, representing parts of the dorsolateral and medial prefrontal cortex-, the posterior cingulated gyrus and the angular gyrus [17]. Furthermore Greene, Nystrom et al. (2004) demonstrated increased bilateral activity in the anterior DLPFC for the judgment of difficult personal moral dilemmas; brain areas associated with abstract reasoning and cognitive control. This brain region also showed an increased activity when participants judged personal moral dilemmas in an utilitarian way (save and reprieve as many people as possible by killing or harming one person) compared to prefer non-utilitarian judges (forsake several people to save one person). Accordingly, neurophysiological and behavioral evidence indicate that utilitarian judgments are preferentially supported by controlled cognitive processes mediated by the DLPFCs. However, the underlying studies are correlational in nature and do not allow a direct causal inference. In contrast, direct modulation of circumscribed brain areas by non-invasive electrical stimulation facilitates the assessment of such causal relations [19]. Direct modulation of the neuronal reactivity of the DLPFC and its impact on human moral reasoning provides an opportunity to establish the missing causal relationship, as electrostimulation techniques like transcranial magnetic stimulation (TMS) and transcranial direct current stimulation (tDCS), in general, may help to establish the causal relationship between brain states and behavior [20]. TDCS as a particular application of transcranial electrostimulation is a non-invasive brain stimulation method to produce a change of neural activity of certain brain regions [21]. Generally, it is assumed that anodal stimulation induces depolarization, thereby increasing spontaneous firing rate and the excitability of cortical neurons. Cathodal stimulation causes an opposite effect, a hyperpolarisation characterized by a decrease of spontaneous firing rate and an inhibition of cortical excitability [22]. Several studies provide evidence for anodal stimulation enhancing motor, perceptual and cognitive functions (for an overview see Nitsche, Cohen [23]). Thus tDCS seems to be a powerful tool to investigate whether a certain brain activity is closely involved in the implementation of certain actions [20]. The possibility to change moral behavior by brain stimulation has been demonstrated via repetitive TMS. Young, Camprodon [24] demonstrated that when judging about the permissibility of an action an interruption of the right temporoparietal junction induced an attentional shift towards the outcome rather than to the underlying action per se [24]. Furthermore, interruption of the right DLPFC can increase the probability of utilitarian responses during evaluation of moral dilemmas [25] and alter fairness-related behavior in an ultimatum game [26]. Recently, it has been demonstrated that TMS- induced disruption of the right DLPFC selectively affected the moral judgment of personal dilemmas whereas TMS of the temporoparietal junction changed moral decisions in impersonal dilemmas [27]

Whereas fMRI studies point to a bilateral involvement of the DLPFC in moral reasoning, neuromodulatory studies primarily investigated the role of the right DLPFC during the judgment of moral dilemmas. The aim of the present study was to test whether neuromodulation of the left DLPFC by means of active tDCS can influence the process of moral reasoning, and in consequence, to demonstrate a causal relation between left DLPFC reactivity and moral reasoning. We used a moral judgment task in which participants had to rate the appropriateness of moral personal dilemma situations. Based on previous results we assumed that modulation of DLPFC activity will impact the moral judgment of personal dilemmas.

## Material and Methods

### Participants

Fifty-four healthy subjects (24 females) participated in the study. All of them signed informed consent prior to the study and affirmed to have no neurological or psychiatric diseases. Participants were naive of the aim of the study and stimulation conditions. They were randomly assigned 2 experimental groups, an anodal (mean age 24.6 ± 3.4, 12 females) and a cathodal (mean age 24.9 ± 4.1, 12 female) stimulation group, matched for age and gender. All subjects underwent 2 stimulation sessions, an active stimulation and a sham stimulation session while evaluating moral dilemmas. For both groups, the order of stimulation sessions (active, sham) was randomized across subjects. Ethical approval for all procedures was obtained prior to the study from the ethics committee of the University Magdeburg and all participants gave written informed consent before participation.

### tDCS

For tDCS application, the active electrode was placed over the left DLPFC corresponding to the F3 electrode of the 10–20 EEG system. The reference electrode was placed over the right parietal cortex, respectively P4. The stimulation was delivered by a battery driven constant current stimulator (NeuroConn GmbH, Ilmenau, Germany) equipped with a pair of rectangular rubber electrodes (5 x 7 cm) covered with saline-soaked sponges. To ensure stable stimulation effects according to Nitsche, Cohen [23], each participant received 10 minutes of tDC-stimulation before starting with the moral judgment task, with 2 mA and 5 seconds fade-in time. After these initial 10 minutes the stimulation continued and participants rated the personal moral dilemmas. The maximum stimulation time did not exceed 20min. These stimulation parameters are considered to be safe [22]. For sham stimulation the electrode arrangement was identical to active stimulation but the stimulator was turned off after 30 seconds with 5 seconds fade-out time. A debriefing after each session revealed that all subjects were unaware of the stimulation conditions. Individual sessions were separated by a minimum period of 5 days and a maximum period of 7 days.

### Experimental task

Participants evaluated 11 moral personal dilemmas (adapted from Greene et al. 2004 [18]). In all dilemmas participants faced a conflict between two opposing moral values or requirements. There was always one option resulting in death or harm for one person while several others could survive and/or escape, and an alternative option that led to death or harm for several people while allowing one person to survive and/or escape the situation. Participants read the dilemmas and rated the appropriateness of the utilitarian action (to save as many lives as possible by directly harming or killing a person) by marking their evaluation on a visual analog scale (VAS). The VAS was bounded by the both extremes *absolute appropriate* and *absolute inappropriate*. The direction of boundaries (either at the left or right end of the scale) was counterbalanced across subjects and the order of dilemmas was varied intra-individually between sham and active sessions.

### Analysis

Statistical analysis was performed by using IBM SPSS Statistics 21. The answers of the dilemma-task on the VAS were measured and averaged. For each participant and each dilemma the mean distance on the VAS from the inappropriate boundary was measured, normalized to the indivdiual value during sham stimulaton and entered into a repeated-measures ANOVA with the within-subject factor *stimulation* (sham/active) and a between-subject factor *group* (anodal/cathodal). In a next step we used a paired t-test to determine the effect of stimulation seperately. Furthermore, to control for potential group differeneces during sham stimulation, we used indepoendent t-test to compared values during sham stimulation between groups.

## Results

**Fig 1:**
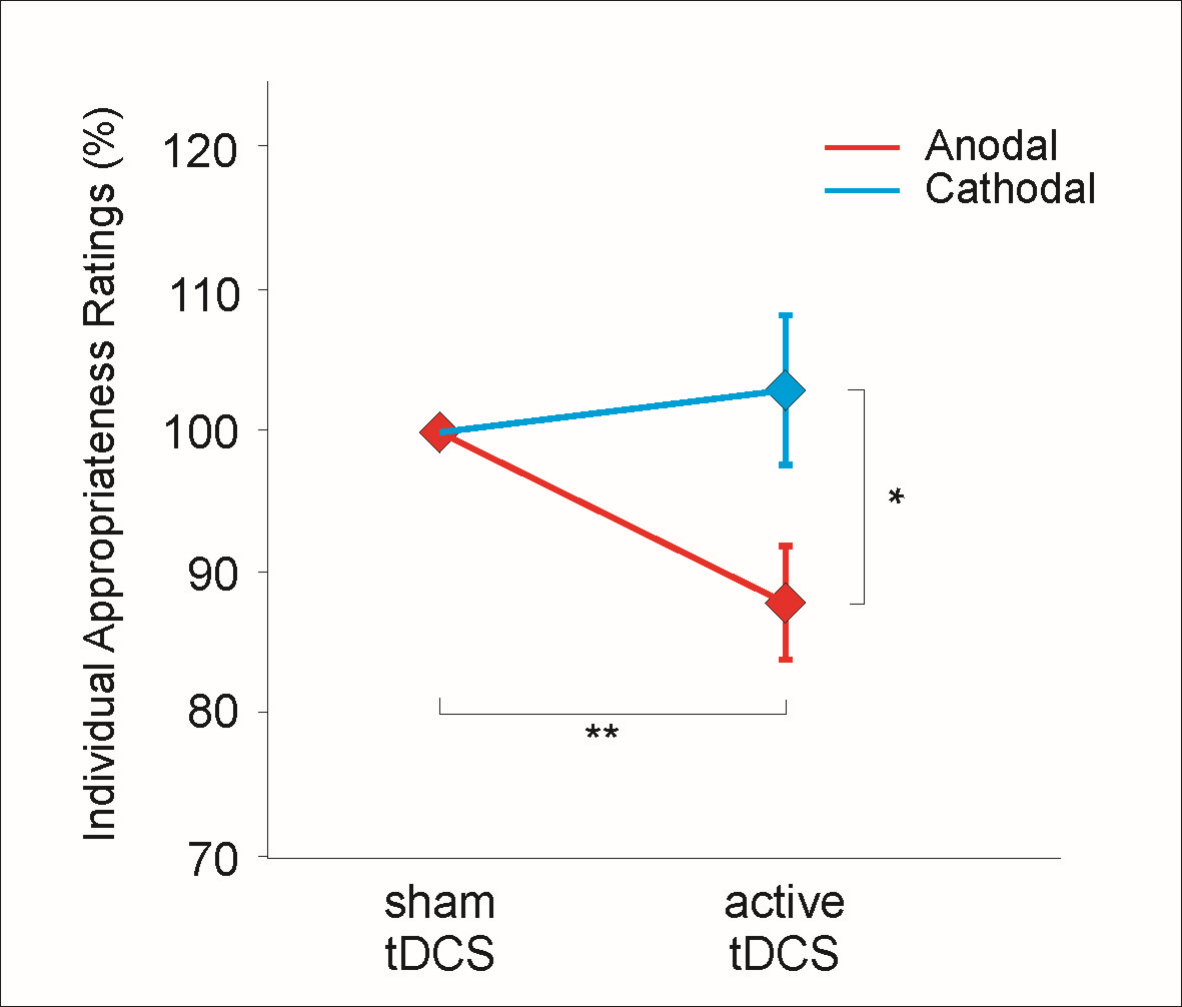
Performance data. Individual Appropriateness Ratings for anodal tDCS and cathodal tDCS over the left DLPFC. There is a decrease in individual appropriateness ratings from sham to active stimulation in the subjects that received anodal tDCS (red line) but not in the subjects that received cathodal tDCS (blue line). Data are normalized to the value during sham stimulation. Asterisks indicate statistical significance. Data are the means +/- SEM.

Independent sample t-test showed that appropriateness rating values (distance in mm on the VAS from the inappropriate to the appropriate boundary) for sham in the cathodal (mean +/- SEM: 54,7 +/-3,8) group did not differ from ratings during sham in the anodal (mean +/- SEM d: 56,6 +/-3,9 mm) group (T(52) = 1.31, p = 0.197). The repeated-measures ANOVA with the within-subject factor *stimulation* (sham/active) and the between-subject factor *group* (anodal/cathodal) revealed a significant *session x group* interaction (F(1,52) = 5.18, p<0.05) and a significant main effect for the factor *group* (F(1,52) = 5.18, p<0.05), but no significant main effect of the factor *stimulation* (F(1,52) = 1.82, p = 0.183) (Fig 1). Subsequent paired t-tests comparing sham and active tDCS for each stimulation group separately revealed a significant stimulation effect for the anodal (T(26) = -2.96, p<0.01) but not the cathodal group (T(26) = -0.58, p = 0.56). From sham- to—stimulation, the mean appropriateness rating was attenuated by 12% in the anodal group. Furthermore, two-sample t-test revealed significant different ratings between the two groups in the active tDCS condition (T(52) = -2,28, p<0.05). Accordingly, during anodal stimulation of the left DLPFC participants rated the utilitarian actions as more inappropriate than they did during sham and cathodal stimulation. Thus, anodal tDCS of the left DLPFC resulted in a shift of preference from an utilitarian, active decisions (i.e. to actively hazard another person’s life to rescue the lives of several people) to non-utilitarian, passive decisions (i.e. to avoid harming another person, but in consequence to accept the harm to several people.

## Discussion

Moral reasoning is a key capacity for human social cognition. In everyday life, people are confronted with several moral dilemma situations in which they have to make decisions based on their moral principles. By the process of moral reasoning, people determine the rightness or falseness of the related actions. These resulting moral judgments can be considered as the foundation of human coexistence and its underlying process seems to be a unique human capacity. The present study shows that such moral reasoning is not a permanent individual entity but can be manipulated by external stimulation of the human brain. Our results demonstrate that the modulation of the left DLPFC reactivity can influence moral decision-making, thereby supporting a causal link between left DLPFC functioning and the process of moral reasoning. We demonstrated that anodal tDCS of the left DLPFC influences moral judgments of personal dilemmas. Relative to cathodal tDCS and sham stimulation, anodal tDCS shifted the participants´ preference towards non-utilitarian actions by making them more likely to judge utilitarian behavior as more impermissible.

The critical involvement of the DLPFC in moral reasoning and decision making is in good agreement with several imaging studies demonstrating a close relation between DLPFC activity and social behavior, especially in moral decision making [17, 18, 28]. These studies indicate that the DLPFC plays an important role in the regulation of potentially counterproductive emotions in the context of social decision making [28]. In particular, differences in the activation of the DLPFC have been associated with the rejection or acceptance of unfair offers in an ultimate game, implying that the DLPFC acts as a cognitive control center to overcome the strong demand to reject the unfair offer [28]. In the same vein, difficult personal moral dilemmas cause a bilateral increase in activity of brain regions that are in general associated with abstract reasoning and cognitive control. Notably, the DLPFC activity is increased when subjects choose the utilitarian decision for such difficult personal dilemmas [18]. Therefore and analogue to the ultimate game, DLPFC activity is associated with the cognitive control necessary to override the emotional needs to avoid personal moral violations. In consequence a higher amount of cognitive control should be associated with more utilitarian moral judgment behavior. However, in the present study we demonstrated that stimulating the left DLPFC by anodal tDCS, and in consequence increasing its neural reactivity, resulted in less utilitarian moral behavior. According to the notion of a stronger involvement of cognitive control mechanisms mediated by the DLPFC during the evaluation of personal moral dilemmas [18], artificially increased activity within this brain area should, in contrast result in more utilitarian answers. Thus, the present results do not fit with the dual-process theory predicting DLPFC functioning to be associated with cognitive control over emotional aversion. Similar contradicting results have been reported recently after the application of rTMS over the right DLPFC during moral judgment [25]. This study reported increased utilitarian response tendencies after disrupting right DLPFC activity. The authors argue that their results can be seen as direct evidence for the pivotal role of the DLPFC in integrating representational emotions during moral evaluation, rather than coding for rational cognitive control over emotional impulse [25]. Furthermore, they assume that the right DLPFC codes secondary social emotions necessary to implement behavior relying on external guidance [29] and abstract rule processing [30]. In this vein, our results also support the notion of a pivotal role of the DLPFC for the integration of emotional responses generated by appraisal of complex social information [25].

Nevertheless, alternatively also reciprocal inhibitory inter-hemispheric relations should be considered. The neurological regularity in the two hemispheres typically has a mutual inhibitory relation—decreased activity or tone in one hemisphere increases activity or tone of the contralateral one and vice versa. If left-DLPFC is activated by anodal tDCS, its inhibitory effect on the right hemisphere (including right DLPFC) will be strengthened and, in consequence, the functionality of the right DLPFC will be relatively declined. This in turn means that cognitive control as one of the right-frontal functions will also decline. As a result, less utilitarian moral judgment behavior should be induced. Thus, the results of the present study could be related to reciprocal inhibitory inter-hemispheric relations and, in turn, support the dual-process theory predicting right DLPFC functioning to be associated with cognitive control over emotional aversion.

Moreover, the mode of tDCS-action should be considered. Generally, it is assumed that anodal tDCS has an excitatory effect on the local cerebral cortex, while cathodal tDCS decreases the cortical excitability in the region under the electrode. However, several recent data demonstrate an opposite anodal/cathodal dichotomy, with e.g. decreased reactivity of specific brain regions after anodal [31] and increased reactivity after cathodal stimulation [32]. The anodal-excitation and cathodal-inhibition effect seems to be valid for the stimulation of the motor and sensory cortex while there is a lot of heterogeneity in cognitive studies [33]. Furthermore, it has been assumed that in an optimal and unaffected level of neuronal reactivity, both an increase as well as decrease of neuronal reactivity will deteriorate the processing of this cortical area. Such inverted U-shape relation has been demonstrated for the influences of tDCS on the auditory cortex [34] and reported for the influence of psychotropic drugs and tDCS effects [35]. Recently, Krause, Marquez-Ruiz [36] captured this scheme for clinical administration of tDCS. Thus, an artificially enhanced neural excitability does not increase performance per se.

Altogether, and with all due caution about interpreting the exact mechanisms underlying cognitive control functioning of the DLPFC during moral reasoning, our study clearly demonstrates that the DLPFC is involved in the judgment of personal moral dilemmas. Moreover, our results do not only indicate that the left DLPFC is an important component within the process of moral judging and that the process of moral judging can be changed online by tDCS, they also evidence a causal relation of DLPFC activity and the process of moral judging. To our knowledge this is the first study that examined the function of the left DLPFC concerning moral decision making by the mean of transcranial direct current stimulation.

## Supporting Information

S1 Table (DOCX)
