# Supplementary material for: Transcranial Direct Current Stimulation of the Left Dorsolateral Prefrontal Cortex Shifts Preference of Moral Judgments
Source: PLoS One. 2015 May 18;10(5):e0127061. doi: 10.1371/journal.pone.0127061 (PMC4436128; doi:10.1371/journal.pone.0127061)
Supplement: S1 Table — (DOCX) [file pone.0127061.s001.docx]

| Subjects  # | | ANODAL  Sham % | Group  Stim % |  | CATHODAL  Sham % | Group  Stim % |
| --- | --- | --- | --- | --- | --- | --- |
| 1 | 100 | | 64,24 |  | 100 | 90,6 |
| 2 | 100 | | 101,69 |  | 100 | 89,45 |
| 3 | 100 | | 92,15 |  | 100 | 81,46 |
| 4 | 100 | | 90,8 |  | 100 | 78,23 |
| 5 | 100 | | 88,53 |  | 100 | 77,58 |
| 6 | 100 | | 111,36 |  | 100 | 89,63 |
| 7 | 100 | | 39,13 |  | 100 | 100 |
| 8 | 100 | | 118,23 |  | 100 | 102,08 |
| 9 | 100 | | 103,42 |  | 100 | 102,41 |
| 10 | 100 | | 111,38 |  | 100 | 119,67 |
| 11 | 100 | | 77,98 |  | 100 | 123,42 |
| 12 | 100 | | 86,83 |  | 100 | 100 |
| 13 | 100 | | 96,94 |  | 100 | 102,78 |
| 14 | 100 | | 42,29 |  | 100 | 179,36 |
| 15 | 100 | | 84,36 |  | 100 | 94,13 |
| 16 | 100 | | 91,75 |  | 100 | 114,23 |
| 17 | 100 | | 106,82 |  | 100 | 47,03 |
| 18 | 100 | | 70,34 |  | 100 | 90,3 |
| 19 | 100 | | 76,42 |  | 100 | 123,03 |
| 20 | 100 | | 95,38 |  | 100 | 164,36 |
| 21 | 100 | | 41,35 |  | 100 | 113,39 |
| 22 | 100 | | 102,36 |  | 100 | 84,76 |
| 23 | 100 | | 103,93 |  | 100 | 78,07 |
| 24 | 100 | | 107,32 |  | 100 | 91,89 |
| 25 | 100 | | 89,46 |  | 100 | 112,01 |
| 26 | 100 | | 87,02 |  | 100 | 143,14 |
| 27 | 100 | | 92,71 |  | 100 | 90,13 |
|  |  | |  |  |  |  |
| Mean | 100 | | 87,93296296 |  | 100 | 103,0792593 |
